# Heart Failure and Cardiomyopathies: CT and MR from Basics to Advanced Imaging

**DOI:** 10.3390/diagnostics12102298

**Published:** 2022-09-23

**Authors:** Pierpaolo Palumbo, Ester Cannizzaro, Maria Michela Palumbo, Annamaria Di Cesare, Federico Bruno, Chiara Acanfora, Antonella Arceri, Laura Evangelista, Francesco Arrigoni, Francesca Grassi, Roberta Grassi, Silvia Pradella, Vittorio Miele, Andrea Giovagnoni, Alessandra Splendiani, Antonio Barile, Carlo Masciocchi, Ernesto Di Cesare

**Affiliations:** 1 Department of Diagnostic Imaging, Area of Cardiovascular and Interventional Imaging, Abruzzo Health Unit 1, Via Saragat, Località Campo di Pile, 67100 L’Aquila, Italy; 2 SIRM Foundation, Italian Society of Medical and Interventional Radiology (SIRM), 20122 Milan, Italy; 3 Department of Anesthesiology and Intensive Care Medicine, Fondazione Policlinico Universitario A. Gemelli IRCCS, Catholic University of the Sacred Heart, 00168 Rome, Italy; 4 Ospedale “Infermi” di Rimini, Viale Luigi Settembrini, 2, 47923 Rimini, Italy; 5 Department of Applied Clinical Sciences and Biotechnology, University of L’Aquila, Via Vetoio 1, 67100 L’Aquila, Italy; 6 Department of Radiology, Università degli Studi della Campania “Luigi Vanvitelli”, 80127 Naples, Italy; 7 Department of Radiology, Careggi University Hospital, Largo Brambilla 3, 50134 Florence, Italy; 8 Department of Radiology, Azienda Ospedaliero-Universitaria, Ospedale Riuniti di Ancona, Via Conca 71, Torrette, 60126 Ancona, Italy; 9 Department of Life, Health and Environmental Sciences, University of L’Aquila, Piazzale Salvatore Tommasi 1, 67100 L’Aquila, Italy

**Keywords:** heart failure, advanced cardiac imaging, cardiomyopathies, cardiac magnetic resonance, computed tomography, CT, CCTA

## Abstract

Since 1997, heart failure (HF) has been designated as a new epidemic. However, it is not easy to find a proper definition since different descriptors are used in clinical practice. Moreover, HF is not a single clinical entity, and there is a close relationship between HF and all cardiomyopathies (CMs). This leads us to also consider accuracy in the characterization of CMs, which is essential to define the therapeutic process of HF patients. This narrative review aims to describe the main mechanisms leading to HF in different CMs, as well as the current diagnostic and prognostic advantages deriving from advanced imaging in the cardiac field.

## 1. Introduction

Since 1997, heart failure (HF) has been designated as a new epidemic, due to its increased incidence and long survival rates resulting in high rates of hospitalization for HF symptoms. However, the actual prevalence of HF remains uncertain [1].

In recent years, many authors have reported an overall stability of HF incidence, most likely attributable to the mean between series with decreased incidence (probably due to the effectiveness of preventive therapies) and series with increased incidence (related to preserved ejection fraction—HFpEF—phenotype) [2,3,4,5,6,7,8,9,10,11,12,13,14,15,16,17].

HF-related mortality rate is increasing (some authors report a 5-year mortality of 50%), though with significant differences among race and age. Increased mortality rate from HF also affects the overall mortality rate of cardiovascular diseases (CVD), which has demonstrated a reversal in trend since 2015, in opposition to the decreased mortality rate observed in the 1980s and 2000s [18,19,20,21].

Combining these data, the prevalence of HF is currently increasing by 1–2% points, showing significant differences between age groups, which reaches 4.3% in the 65–70-year range, and with an estimated increase of up to 8.5% in the next few years [22,23].

HF has therefore become a major health problem and its correct definition is mandatory, although many difficulties still remain:

1. *HF is not a single clinical entity*, but it is a complex syndrome theoretically deriving from functional or structural cardiac insults that alter the heart pump. Current criteria combine similar indicators; however, the prevalence of the disease may be underestimated because the diagnosis is accomplished only when the syndrome is clinically evident [24,25,26,27,28].
2. *Different descriptors have been used in recent years to describe HF* (e.g., right, left, or biventricular involvement, cardiac output abnormalities, timing of onset). The most important classification (followed by current guidelines and clinical trials) differentiates between HF with preserved ejection fraction (EF) (HFpEF; i.e., EF > 50%) or reduced EF (HFrEF; i.e., EF < 40%), or with medium involvement of the EF (HFmEF, i.e., EF ranging from 41 to 50%).

This classification is based on the definition of a primarily systolic or diastolic dysfunction. Systolic dysfunction refers to the impairment of myocardial contraction, i.e., loss of inotropy. Consequently, end-systolic volume and preload (to a lesser extent) increase, resulting in a stroke volume decrease. Resultant of these alterations is a reduction in EF. The pressure–volume curve shifts to the right, meaning that no increase in ventricular pressure is required to ensure the same ventricular volumes. In chronic systolic dysfunction, the ESPVR moves to the bottom-right of the graph since higher volumes chronically determine cardiac remodeling to increase myocardial compliance [24].

**Figure 1:**
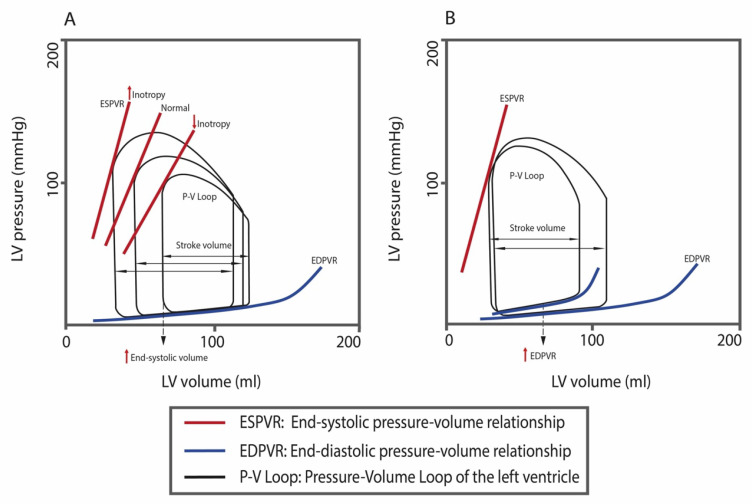
In panel (**A**), systolic dysfunction; in panel (**B**), diastolic dysfunction, with an ideally preserved intrinsic inotropy. Each pressure–volume loop (P–V loop) represents a heartbeat. Panel (**A**): P–V loop diagrams show changes in inotropy with a relative change in the slope of the end-systolic pressure–volume relationship (ESPVR); the decreased inotropy decreases the slope-ESPVR, shifting to the right and downwards, with impaired myocardial contractility, which generates less pressure at a given LV volume. As a consequence, the P–V loop shifts to the right and downwards, with an overall smaller loop area, and with a decrease in stroke volume, which is the width of the P–V loop (and, consequently, a lower ejection fraction), and an increase in end-systolic volume (corresponding to the left lower corner of any P–V loop). Panel (**B**): Reduced myocardial compliance increases the slope of the ventricular end-diastolic pressure–volume relationship (EDPVR), which shifts to the left. The increase in EDPVR will result in a higher filling pressure or end-diastolic pressure in the left ventricle, and a normal or reduced ventricular filling volume (decreased end-diastolic volume). A greater end-diastolic pressure leads to a greater pulmonary capillary wedge pressure. If the end-diastolic volume is decreased for the higher filling pressure, the stroke volume can also decrease per cardiac cycle.

Conversely, in diastolic dysfunction, intrinsic inotropy is preserved while there is an increased stiffness and impaired relaxation of the ventricle, resulting in alteration of the filling pattern. This alteration implies preserved volumes and systolic shortening, compared to an increased ventricular pressure (needed to ensure similar ventricular volumes) due to the higher myocardial stiffness (reduced compliance) [24] (Figure 1).

Differentiating among these patterns remains crucial, also considering that accurate risk stratification is challenging.

The clinical rationale therefore aims to identify the presence and cause of myocardial deficits, as well as of all those structural alterations with a strong clinical impact on the evolution of HF syndrome.

However, different thresholds to differentiate between HFrEF and HFpEF patients have been reported in the literature. In the OPTMIZE-HF and ADHERE trials, a threshold of 40% was used to categorize HF patients; the American Heart Association (AHA) suggests a threshold of 50%, while the European Society of Cardiology (ESC) differentiates in reduced, mild-reduced, and preserved EF phenotype [28,29,30,31,32,33,34,35,36,37,38,39].

This difference has some implications, since an inaccurate measurement, or different thresholds, can obviously lead to different inclusion criteria. In this regard, cardiac magnetic resonance (CMR) is the gold standard for defining cardiac volumes and function, even though its role in defining HFpEF patients remains uncertain.

Moreover, different studies have demonstrated that mild and reduced phenotypes derive similar benefits from similar therapeutic treatments, suggesting that they may belong to a similar category [28,29,40,41].

3. *HF may offer different scenarios*: (i) Chronic worsening; (ii) new onset; (iii) advanced HF, needing advanced medical therapies such as heart transplantation and mechanical ventricular assistance. Identifying acute HF is crucial to adequately measure the real burden of the disease [3,8].

## 2. CMR and CCTA Imaging

Transthoracic echocardiography (TTE) plays a pivotal role in the definition of HF [29]. Thanks to its lower costs and availability, this technique allows the assessment of systolic and diastolic function.

However, TTE is not adequate in tissue characterization, and alteration in myocardial echogenicity results as a poor surrogate.

In recent years, many authors have emphasized the role of CMR and coronary computed tomography angiography (CCTA) in the management of CV disease, also in an emergency setting [42,43,44,45,46,47,48].

1. CMR allows a holistic approach to cardiac patients.

This technique (i) properly describes the heart’s interaction with other mediastinal structures, allowing identification of situs inversus or pectus excavatum; (ii) offers a detailed depiction of cardiac morphology, myocardial function, and perfusion pattern, which help discriminate among phenotypic presentation of different cardiomyopathies (CMs) [49]; (iii) shows the intravascular flows, with 4D flow technique, which also allows the vectorial patterns of the flow to be decomposed [50].

The net effect is an in vivo virtual model of the heart obtained through a multiparametric disease-oriented approach to the heart patient.

CMR offers many advantages in defining cardiac function and structural HF-related changes [51,52,53].

CMR-derived parameters are reproducible and accurate also in HFpEF, and allow underlying conditions with insidious clinical onset to be uncovered [54].

**Figure 2:**
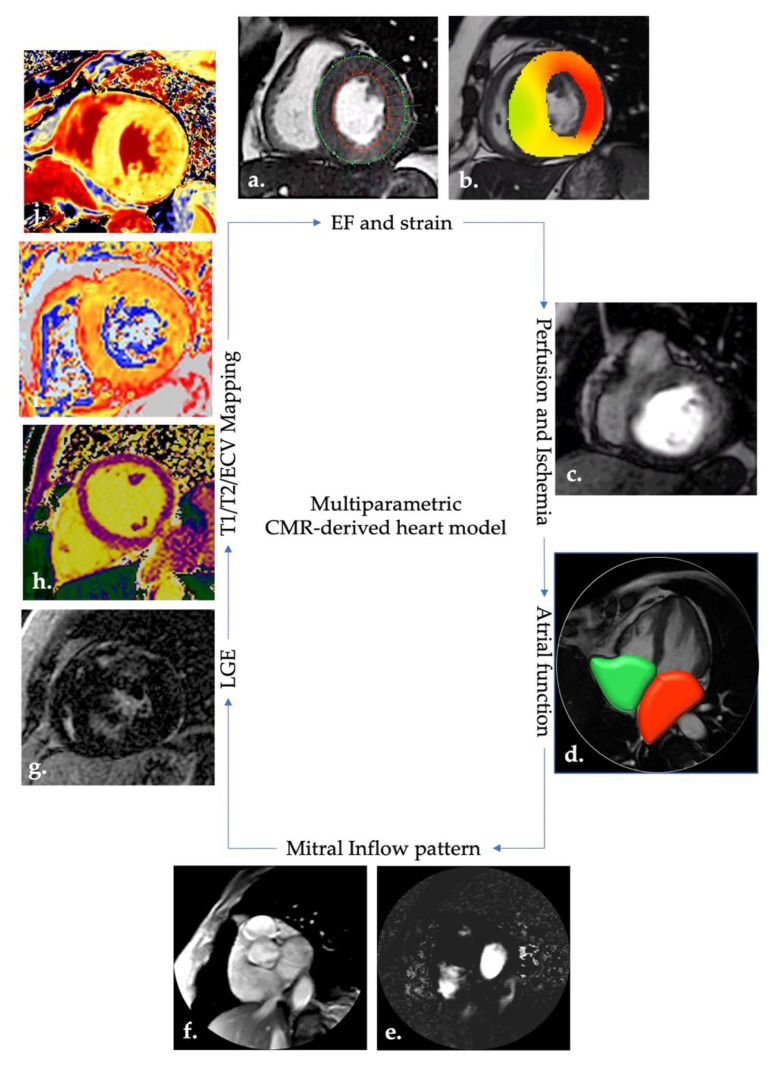
CMR heart model using different imaging biomarkers. Images (**a**–**j**) show different examples of CMR biomarkers useful in a proper definition of a HF patient. Images (**a**,**b**) show cine images; in image (**a**): endocardial (red) and epicardial (green) contours are drawn in all SA stacks, both in diastole and systole through a dedicated software; endocardial and epicardial contouring are used to estimate ventricular volumes and myocardial mass, respectively. In image (**b**): an example of radial strain depicted by a colorimetric map superimposed on an SA cine image; colorimetric map shows the degree of myocardial deformation evaluated by the strain analysis. Bright colors suggest a lower deformation. Image (**c**) shows an example of first-pass perfusion imaging: the wide hypointense rhyme within the inferior wall represent a region of myocardial ischemia. Image (**d**), atrial evaluation: atrial volumes allow dimension and function to be estimated: left and right atria are showed in the figure through red and green reconstruction in systole (LA and RA maximum volume). Images (**e**) (phase image) and (**f**) (magnitude image) show an example of phase-contrast sequence for the evaluation of valvular flow. Images (**g**–**j**) show the sequence currently adopted for proper tissue characterization. In image (**g**), the post-contrast LGE sequence shows a faded enhancement within hypertrophic segments in an HCM patient. Images (**h**–**j**) show mapping sequences. In image (**h**), regular naïve (pre-contrast) T1 mapping in a young athlete. In image (**i**), abnormal T2 mapping (high values) in non-treated AL amyloidosis. In image (**j**), abnormal ECV sequence (high values in lateral wall) in Anderson–Fabry disease. CMR: cardiac magnetic resonance; HF: heart failure; SA: short axis; LA: left atrium; RA: right atrium; LGE: late gadolinium enhancement; HCM: hypertrophic cardiomyopathy; AL: amyloid light chain; ECV: extracellular volume.

Ventricular volumes, myocardial deformability by strain analysis, tissue characterization and myocardial mapping, mitral inflow pattern, left atrial volumes and first-pass perfusion imaging allow an accurate detection of HF markers, while myocardial tagging, also used to assess myocardial deformability by means of additional sequences, now seems to be outdated by the widely demonstrated validity of the strain (Figure 2).

Moreover, CMR has become the imaging method of choice to assess myocardial fibrosis thanks to the uncontestable advantages deriving from CMR in accurate tissue characterization [55,56,57,58,59,60,61,62,63,64,65,66,67].

With these premises, CMR, like TTE, is highly recommended in patients with HF and a suspected CM, or in those with a poor acoustic window [29].

The main limits of CMR lie in time-consuming acquisitions, claustrophobia and the presence of a metal device, although, in recent years, this is becoming a marginal problem given the increasingly widespread use of safety devices compatible with MR systems as well as the optimization of scanners to improve image quality in the presence of metallic prosthesis [68,69].

2. CCTA is accurate in describing coronary anatomy and in the approach to coronary artery disease (CAD) [70,71,72].

Great advantages derive from stratification of disease severity in terms of: (i) degree of stenosis; (ii) CAD extension; (iii) plaque phenotype.

From the study of Motoyama et al., CT-derived markers of plaque vulnerability (i.e., low density, spotty calcification, napkin sign, coronary remodeling, and coronary contrast drop) show a relevant impact in disease progression, with a high predictive value for major cardiac events [73,74].

Plaque phenotype and CAD extension seem to be highly involved in disease progression, since disease severity in terms of degree of stenosis is not the only factor which determines ischemia [75].

Moreover, although different ischemic surrogates from conventional analysis have recently been provided, CCTA and conventional analysis do not help identify the main myocardial injurer [76], but the availability of different ischemic surrogates [77]. Therefore, CCTA plays only a marginal role in high-risk patients [78].

The IMAGE-HF 1C trial demonstrates no significant difference in the management of patients with HF of unknown etiology using invasive coronary angiography (ICA) and CT [79].

The current guidelines recommend CCTA as a “should be” technique in patients with HF and low to intermediate pre-test probability of CAD or those with equivocal non-invasive stress tests, to rule out coronary artery stenosis (class IIa) [29].

**Figure 3:**
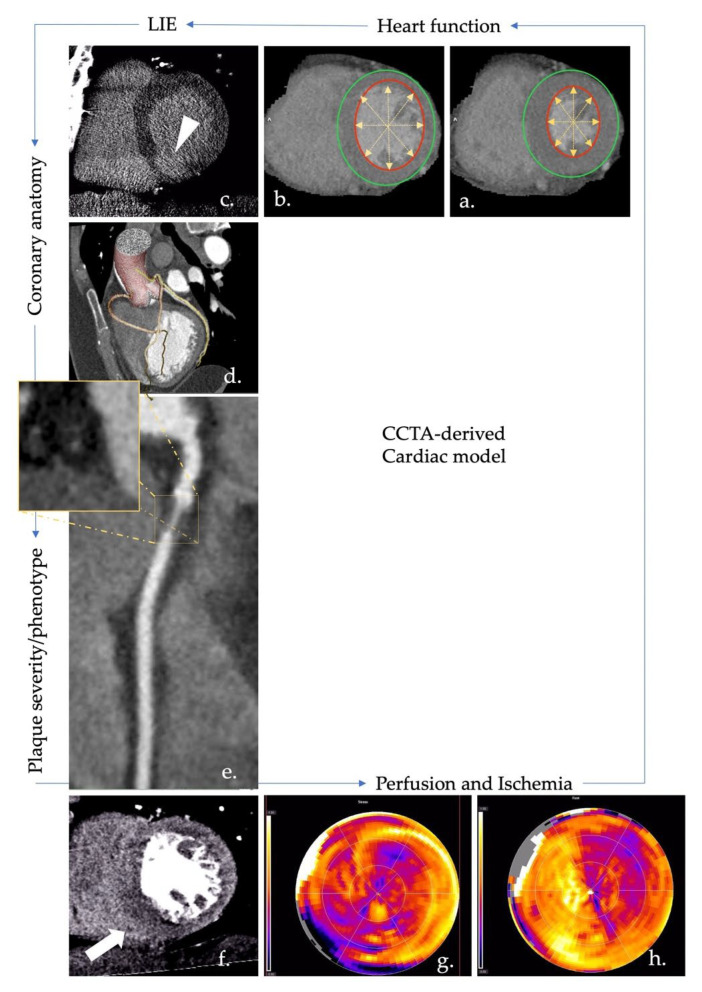
CCTA biomarkers useful for a proper definition of HF patient. Typically, CCTA is a well-known tool for description of cardiac anatomy; in image (**d**), volume-rendered reconstruction of aortic root and coronary anatomy superimposed onto an MIP reconstruction of cardiac chambers in a similar projection. CCTA allows (i) an accurate detection of coronary stenosis; (ii) a good definition of the degree of stenosis; (iii) an optimal definition of plaque phenotype. In image (**e**), a severe non-calcified stenosis in proximal RCA; the plaque shows a prevalent lipidic composition. High-temporal resolution exhibited by the latest CT scanners also allows the complete cardiac cycle with a relatively low-dose exposure to be acquired; the retrospective analysis could be useful for the estimation of cardiac volumes and function. Images (**a**,**b**) show the SA view of the same plane in diastolic and systolic phase: dedicated software can automatically derive volume and function from the contouring of the entire chamber, similar to CMR. Using vasodilator agents, CCTA also allows the evaluation of myocardial perfusion, both with static or dynamic acquisition, mainly depending on the technology of the scanner. Images (**f**–**h**) show a stress acquisition (image (**f**)) and colorimetric map of stress and rest acquisitions (images (**g**,**h**), respectively). As highlighted by the white arrow, reversible ischemia is detected in the inferoseptal wall on the basal and medial plane. Lately, great interest has also been derived from tissue characterization, although mainly through the use of dual-energy, which allows the definition of an iodine map. In image (**c**), an example of LIE with late acquisition revealing a subendocardial iodine enhancement related to a previous MI. CCTA: coronary computed tomography angiography; HF: heart failure; MIP: multiplanar image projection; RCA: right coronary artery; CT: computed tomography; SA: short axis; CMR: cardiac magnetic resonance; LIE: late iodine enhancement; MI: myocardial infarction.

The main limits lie in radiation exposure and severe kidney failure patients as a contraindication to contrast medium injection. However, low-dose exposure achieved by the latest scanners increases the potential availability of CCTA in HF imaging for both function and tissue characterization assessment [80,81,82,83,84] (Figure 3).

3. Finally, non-invasive stress imaging is class IIb for the study of vitality and ischemia in patients with CAD considered eligible for revascularization [29].

### 2.1. HF Markers and CMR-CCTA Findings

#### 2.1.1. Cardiac Function and Geometry

Among adaptive responses including molecular, metabolic, physiological, and structural mechanisms, the neurohumoral model alone is unable to fully explain the progression of HF. Conversely, the presence of cardiac remodeling is associated with a worse prognosis and a less favorable disease progression.

Cardiac remodeling involves micro- and macroscopic cardiac structures, from cardiomyocytes’ gene expression to changes in the architecture and geometry of cardiac chambers.

These structural adaptative mechanisms are essentially two halves, defined as cardiac hypertrophy and categorized into concentric and eccentric.

These adaptive alterations are determined by the type of myocardial insult and, consequently, of wall stress; therefore, conditions of increased afterload (e.g., hypertension or aortic stenosis) cause pressure overload, and an increased wall thickness is necessary to maintain an adequate wall tension; conversely, conditions of augmentation in preload (e.g., aortic insufficiency or myocardial infarction) increase ventricular diameter with or without reduced wall thickness due to the volume overload, requiring an increased wall tension to keep intraventricular pressure unchanged, which is useful in determining systolic function.

Augmented afterload increases wall thickness (concentric pattern), while conditions of volumetric overload lead to ventricular dilation (eccentric pattern or dilated phenotype, typically observed in patients with HF) [24].

CMR has become the gold standard for measuring ventricular volumes, mass, and EF of both ventricles [85].

In recent years, large international registers have allowed the definition and continuous updating of reference values for morphological and functional CMR parameters [86,87,88,89].

Knowledge of normal values is necessary for the interpretation of all pathological changes.

Essentially, the contouring of endocardial and epicardial layers, processed by CMR analysis software, both in diastolic and systolic phase, allows the definition of cardiac volumes. Cardiac volumes are useful for evaluating ventricular dilation and global systolic performance.

Moreover, other parameters (e.g., sphericity index) that potentially help assess cardiac geometry are included in these registries, offering the possibility to evaluate cardiac dilation beyond volumes [90].

Recently, the introduction of automated analysis based on machine learning has showed a satisfying degree of agreement with the current semiautomatic analysis, thus suggesting the potentiality of using a method independent of the reader’s experience [91]. However, further research is needed to clarify this aspect, given the variabilities potentially deriving from a fully automated analysis, and current reliability highlighted by IA in the imaging field [92,93,94,95].

Use of the latest CT scanners has also led to great interest in CCTA applications for the evaluation of ventricular volumes and function. In fact, CCTA has a higher spatial resolution than CMR and TTE, as well as a comparable temporal resolution. Although there is a need for a retrospective study acquiring the entire cardiac cycle, latest low-dose scanners have demonstrated an ability to assess ventricular function comparable to CMR and greater than TTE, with a radiation dose even lower than the radiation dose administered with prospective acquisition of first-generation scanners [96].

#### 2.1.2. Subclinical Deformation Anomalies (Myocardial Strain)

In recent years, great interest has been generated in strain analysis as a more accurate imaging biomarker of early impairment of global function beyond conventional EF [97].

Strain refers to the dimensionless percentage of myocardial deformation in a specific direction. Myocardial fibers are indeed disposed following two main directions in the myocardium. The longitudinally oriented fibers lie in the subendocardial layer and, to a lesser extent, in the subepicardial layer. Conversely, the circumferentially oriented fibers lie in the mid-myocardial layer.

Three main components are identified, including longitudinal, circumferential, and radial strain, and three shear strains. Shear strains increase contraction and, thus, EF by amplifying the 15% shortening of myocytes into 40% radial LV wall-thickening.

Radial strain is tethered to other longitudinal and circumferential fibers, since no radially oriented fibers are disposed within the myocardium [98].

Evaluation of different strains theoretically allows staging of myocardial injury and disease progression. Early injuries primarily affect longitudinal strain, while circumferential and other shear strains such as torsion could become preserved or even increased as compensating mechanisms. Conversely, transmural injury or, similarly, disease progression also affect other strains resulting, in the end, as decreased EF [99].

Strain may be obtained by processing (also retrospectively) CMR cine sequences regularly included in standard acquisition protocols. Different studies stress the potentiality of strain analysis, although: (i) a higher feasibility seems most related to the level of reader expertise [100]; (ii) different software could be used for strain analysis, producing different values [101]. However, recent software with machine learning implementation could represent a potential aid in reducing interobserver variability [102].

Wide clinical utility derives from strain analysis.

Romano et al., in a study including 1274 patients, showed that patients with global longitudinal strain (GLS) ≥ median (−20%) had significantly reduced event-free survival compared with those with GLS < median, with an incremental 22.8% of death risk for each 1% worsening in GLS (HR: 1.228 per percent; *p* < 0.001) [103].

Similarly, in another study by Romano et al., GLS was confirmed as a powerful independent predictor of mortality in a 1012-patient population with ischemic or nonischemic dilated cardiomyopathy, incremental to common clinical and CMR risk factors including EF and LGE [104].

Additionally, from the study by Houard et al., in a total of 266 patients with HFrEF (mean LVEF 23 ± 7%) submitted to RV function assessment using CMR, 2D RVGLS provided strong additional prognostic value to predict overall and CV mortality (over EF, TAPSE and fractional area change) [105].

Finally, from the PARAMOUNT investigators, in a study population of 219 HFpEF patients, lower longitudinal and circumferential strains were detected compared to the control group with no HF, suggesting a systolic dysfunction despite the preserved EF also as a mechanism of potential disease progression.

Lower GLS was modestly associated with higher NT-proBNP, even after adjustment for 10 baseline covariates including LVEF, measures of diastolic function, and LV filling pressure (multivariable adjusted *p* = 0.001).

However, a LV GLS < 16% has a sensitivity of 62% and a specificity of 56% for the diagnosis of HFpEF by invasive testing [106].

Lastly, from the study of Wang et al., CCTA could also be useful in analyzing strain in HF patients with CCTA-derived 3D-GLS, being reliable and interchangeable compared to CMR strain in quantitatively assessing myocardial mechanical changes in HF patients [107].

#### 2.1.3. Interstitial Matrix, LGE, T1, T2, and ECV Mapping

In HF, the interstitial space includes reparative and interstitial fibrosis. Interstitial matrix and connective tissue play an important role in maintaining ventricular shape. However, different injuries are closely related to pathological changes in interstitial matrix composition. A direct injury determines myocyte necrosis with consequent focalized fibrosis in the site of myocardial damage, by increasing the activity of the metal proteases. Otherwise, such a systemic condition as pressure overload can induce increased activity of fibroblasts (which differently perceive the mechanical forces), leading to an increase in the interstitial fibrotic component. Additionally, the renin–angiotensin–aldosterone system belonging to the neurohumoral system can increase collagen production by stimulating fibroblasts [24].

CMR is recognized as a pivotal method in identifying myocardial fibrosis through late gadolinium enhancement (LGE) sequences [108]. LGE is able to identify the underlying etiology in a substantial proportion of HF patients [109]. In particular, the subendocardial distribution of LGE identifies an ischemic injury as opposed to fibrosis with meso- or subepicardial distribution, typical of non-ischemic alterations [110].

In recent years, widespread interest has also arisen from mapping sequences. Mapping allows a new approach to myocardial characterization, through a point-to-point map of T1 and T2 relaxation times. Mapping indeed offers a quantifiable view of the myocardium, revealing the presence of focal or diffuse fibrosis (not properly assessable with standard sequences), myocardial water content, and quantification of extracellular volume.

Large multicenter studies have emphasized the strong impact that mapping sequences have had in the approach to non-ischemic cardiomyopathy, showing strong predictor validity in survival and HF progression.

From the study by Puntmann et al., T1 mapping reveals a key role in risk assessment of non-ischemic CM (NICM), predicting survival and risk of HF [111].

Extra-cellular volume (ECV), on the other hand, allows the quantification of extracellular matrix (as percentage), whose expansion can result from edema, fibrosis, or storage disease. Moreover, recent evidence suggests that ECV allows the quantification of different myofibrillar and interstitial matrix compositions, which is also useful in the discrimination between different hypertrophic phenotypes [112].

In the study by Vita et al., ECV has shown high capability in predicting the clinical evolution of HF in a patient population of 240 with ICM and NICM [113].

T2 imaging and, in particular, T2 mapping are valuable tools for the quantification of water content. Although high T2 values cannot discriminate between inflammatory and non-inflammatory edema, T2 is helpful in defining acute myocardial injury, more aggressive storage disease and congestive water accumulation in advanced HF [114,115].

The current role of CCTA in tissue characterization deserves specific mention. The high temporal resolution of CCTA with dual energy and recent spectroscopic application and iodine mapping have increased fascination of CCTA in tissue characterization, with dual-source CT resulting in some studies comparable to CMR [116,117,118,119,120,121,122].

From the study of Ohta et al., myocardial delayed enhancement (DE) CT enables accurate detection and localization of scarring in patients with HF when compared to CMR-LGE [123]. Similarly, CCTA-ECV from late iodine enhancement DECT is relevant in HF patients, with ECV elevation being an independent risk factor for HF without CAD, also correlating with cardiac structure and function [124].

However, contrasting results were reported in a recent study on DECT; readers’ experience seems to impact the capability of detecting small non-ischemic scars, suggesting a limited routine approach to DECT [110].

#### 2.1.4. Transvalvular Inflow Pattern

Trans-mitral and pulmonary inflow patterns are well-known echocardiographic markers of HF [125,126].

Different studies explored the correlation between CMR and TTE in the evaluation of diastolic flow in a wide range of conditions, ranging from normal to restrictive forms [127]. In conclusion, similar results suggested a potential role of CMR both in confirming TTE findings or as a primary indication for patients not suitable for TTE (i.e., non-adequate acoustic window).

Inflow patterns are obtained through phase contrast (PC) sequences, which quantify the flow in a single plane by identifying each voxel as a flow vector with specific volume and velocity.

However, current CMR protocols do not routinely include PC sequences for mitral inflow pattern evaluation in CM studies.

#### 2.1.5. Left Atrial Volumes and Function

Evaluation of left atrial disease is of primary importance to define the risk of patients with HF, considering that increased atrial size is frequently associated with atrial fibrillation and cardiac outcomes [29,128,129,130].

Atrial size is one of the best indices of atrial disease. A threshold of >32 mL/m^2^ is regularly adopted when considering atrial enlargement, which increases to >40 mL/m^2^ in the presence of atrial fibrillation [29].

Beyond atrial strain, which is still the object of research, particular attention should be paid to atrial volumes and functionality of the left atrium.

Khan et al. studied a large patient population of about 11,000 cases, where a moderate and severe increases in atrial volumes were significant predictors of death and cause of mortality in patients referred for CMR [131].

Although atrial volumes express the maximum atrial volume measured in systole (generally excluding pulmonary veins), atrial function should be evaluated during its phasic contraction. Therefore, three main indices are evaluated: (i) total emptying volume (or atrial stroke volume, as the difference between maximum atrial volume and minimum atrial volume); (ii) passive emptying volume (which refers to the early systolic phase of ventricular filling; defined as difference between maximum volume and atrial pre-contractile volume); (iii) active emptying volume (which refers to the late contractile atrial phase or end-diastolic phase; defined as difference between atrial pre-contractile volume and minimum atrial volume) [132].

In the study by Markman et al., both reduced active and passive emptying volumes were associated with major cardiac events [133].

Moreover, from the Dallas Heart Study, total emptying volume resulted as an independent predictor of mortality, even in the absence of increased atrial volumes [134].

CCTA-derived LA atrial function also showed a high impact in HF prediction. From the study of Lessick et al., including 788 consecutive patients with normal sinus rhythm who had undergone spiral CT scans, CCTA-derived LA function was shown to be a strong predictor of HF hospitalization or CV death, independent of clinical risk factors, LA volume, and LV systolic function [135].

#### 2.1.6. Ischemia

Ischemia is one of the main determinants of HF progression and one of the main markers to identify in cardiac patients with an intermediate-to-high pre-test risk for ischemic heart disease. Even the pathophysiological process underlying the development of HF in ischemic patients can be variable.

Patients with an obstructive epicardial stenosis are more prone to developing HFrEF as a consequence of an acute myocardial infarction (AMI).

Conversely, in HFpEF models, onset of coronary microvascular dysfunctions (CMD) in non-infarcted areas contributes to the recurrence of ischemia and progression of organ dysfunction, thus determining the onset of HF symptoms, even in the absence of reduced systolic function [136].

From the CE-MARC 2 coronary physiology sub-study, high incidence of CMD could also be found in patients with non-obstructive CAD [137].

Moreover, from the CorMicA trial, identification of ischemic substrate in non-obstructive CAD, more likely due to microvascular injury, is of primary importance considering that standardized approaches often fail in the correct management of CMD patients [138,139].

CMR showed high accuracy in rule-in ischemia, with up to 94% accuracy compared to the gold standard invasive fractional flow reserve (FFR) [140,141].

Different stressors may be used to perform stress CMR, including vasodilators (which induce a “steal phenomenon” and loss of autoregulation mechanisms, leading to perfusion defects) and inotropic agents (which increase myocardial metabolism with an ischemic effect).

In recent years, CCTA has also shown encouraging results in the study of the myocardium and perfusion patterns, thus appearing useful in the approach to CMs, especially to ischemic heart disease [116,117,118,119,120,121,122].

Dynamic and static acquisition can be performed for CT myocardial perfusion imaging (MPI), although only dynamic acquisition allows myocardial blood flow to be quantified and is more accurate. Considering that scan timing is crucial for static acquisition, and many factors could influence optimal scan time, prospective estimation of optimal scan timing remains challenging.

From the study by Takx et al., the diagnostic validity of CT-MPI is comparable to other perfusion techniques [142].

Moreover, from the SPECIFIC multicenter study, the clinical utility of CT-MPI in discriminating the functional significance of CAD increases when CT-MPI is complementary to CCTA or FFR-CT [118,120].

#### 2.1.7. Lung Involvement

Non-cardiac CT is important in accurately staging lung involvement in HF.

Lung dysfunction is indeed common in patients with HF because reduced cardiac pump capacity may cause slight to severe lung edema, with pleural effusion, increased blood volume, and consequent reduction in ventilation space [24].

When vascular congestion develops, lung compliance is reduced and respiratory work increases. Pulmonary edema can show different degrees of lung involvement, ranging from a smooth thickening of the interlobular septa up to the filling of the alveolus in the advanced stage.

Non-cardiac CT is still a powerful imaging tool in detecting lung injury in several diseases and, recently, CT was highly impactful during the worldwide pandemic [47,83,143,144,145,146,147,148,149,150,151,152,153,154,155,156,157].

In HF also, CT scans can identify both vascular congestion and interstitial–alveolar involvement, allowing, in many cases, a discrimination from interstitial involvement of an inflammatory pattern or other interstitial pneumoniae [158].

## 3. Cardiomyopathies (CMs) and HF

CMs are highly heterogeneous both in presentation and in response to treatment; in this regard, in the HFrEF phenotype, therapies are standardized regardless of what CM phenotype subtends the HF; conversely, in the HFpEF phenotype, only specific therapies may positively impact the outcomes of the disease [159].

The close relationship existing between HF and CMs, therefore, leads researchers to consider the accurate characterization of CMs as essential in defining the therapeutic process of HF patients.

Among all CMs, dilated (DCM), hypertrophic (HCM) and restrictive (RCM) CMs show a high correlation with HF, while arrhythmogenic CM shows a primary arrhythmic presentation and rarely an evolution toward HF [160].

In recent years, advanced cardiac imaging has taken on an increasingly important role in CM characterization, although suitability criteria and a technical basis remain the cornerstone [44,52,53,161,162].

### 3.1. Ischemic Heart Disease (IHD)

Approximately 70% of HF cases have been attributed to IHD in some major clinical trials [163].

From the SOLVD study, IHD tended to have a greater impact than NICM, with double the risk of hospitalization and quadruple the risk of death [164].

IHD includes a wide spectrum of clinical manifestations, and a chronic clinical scenario known as chronic coronary syndrome (CCS) has recently been introduced to differentiate a clinical stable presentation from an acute one (acute coronary syndrome or ACS). Different outcomes, however, may result from the dynamic nature of IHD, and ACS can also destabilize a long-standing apparently stable clinical scenario [78].

The ISCHEMIA trial has recently discovered that a non-superior benefit derives from an initial invasive vs. conventional medical treatment in cases of moderate-to-severe ischemia, especially in the early time-window of observation and in patients with good systolic performance [76,165,166].

In this regard, from the extended STICHES, revascularization strategies plus medical therapies seem more effective than medical therapy alone in the treatment of patients with reduced EF, thus suggesting a real benefit to coronary revascularization in patients with both ischemia and HFrEF [167,168].

#### 3.1.1. Acute Coronary Syndrome

ACS is often associated with intracoronary thrombosis, although recently, beyond typical obstructive disease, a non-obstructive presentation known as MINOCA (myocardial infarction with non-obstructive CAD) has been introduced, potentially determined by a vasospasm or microvascular dysfunction, although a self-lysed or distal thrombosis cannot be excluded [169].

Actually, MINOCA represents a conundrum resulting from very different syndromes, accounting for a considerable portion of ACS, ranging from 7 to 15% of cases [170]. Clarify the underlying specific mechanism determining the MINOCA is mandatory for tailoring secondary prevention measures aimed at improving the overall prognosis of ACS [171,172]. In this regard, from data provided by Dastidar et al., CMR established a definitive diagnosis in 70% of a patient population, including 204 consecutive patients classified as MINOCA, thus showing a relevant impact in MINOCA management [173].

Acute injury causes direct damage to myocytes with consequent fibrotic replacement in areas of necrosis. The activation of the neurohumoral systems also leads to an increase in adverse remodeling of the left ventricle, leading to dilation and dysfunction of both infarcted and non-infarcted myocardium up to HFrEF. From the SOLVD and SAVE studies, therapies with ACE inhibitors, which act on the neurohumoral system, can modify the risk of developing HF [136,164,174,175].

Another important factor is post-infarction inflammation.

Although inflammation is a process that plays a pivotal role in post-infarct myocardial healing, excessive inflammation seems to be involved in the development of ventricular remodeling. Excessive inflammation results in higher inflammatory cellular infiltration within the infarcted area, which causes direct (through phagocytic activity) and indirect (through secretory activity, which also involves the healthy myocardium) damage, up to the so-called infarct expansion. Thus, higher inflammatory response results in worse remodeling, up to aneurysmal dilatation, and HF development. In this regard, the extent of the infarction seems to be linked to higher inflammatory response [176].

From the study by Suleiman et al., patients with increased post-infarction C-reactive protein had an increased risk of death or HF, with the risk being proportional to the level of C-reactive proteins [177].

The ambivalence of inflammation as a dual healing/damage modulator translates into different responses to inflammatory therapy. In fact, steroidal and non-steroidal anti-inflammatory therapies from the early post-MI phase can worsen the infarction, preventing normal healing processes and increasing the chances of rupture and infarct extension [178,179,180]. On the contrary, from a recent study by Hafezi-Moghadam et al., steroid anti-inflammatory therapy administered before the acute MI phase would seem to be protective [181].

This evidence suggests that inflammatory modulation is a potential target therapy in AMI to reduce cardiac remodeling and improve ventricular function, as demonstrated from the VCU-ART pilot study through the administration of anakinra1 (an IL-1 receptor inhibitor) in patients with recent STEMI and as a consequence of CRP level reduction [182].

#### 3.1.2. Chronic Coronary Syndrome (CCS)

CCS identifies patients with an apparent stable CAD disease, in whom the integrity of the myocardium can be compromised by the deleterious effects of chronic ischemia. Chronic ischemia is a main determinant of diastolic dysfunction inducing myocardial stiffness and altered myocardial relaxation. Diastolic dysfunction can limit blood flow, thus increasing ischemia, leading to congestion and dyspnea, which are typical symptoms of HF, even with a preserved EF.

Different factors can also induce chronic ischemia: (i) Systemic inflammation, which can lead to myocardial and microvascular vulnerability as well as to reduced perfusion and ischemia when the myocardium increases its metabolism. In fact, all conditions altering systemic inflammatory response can also result in macrophage infiltration and interstitial fibrosis, leading to ventricular dysfunction and remodeling. (ii) Increased afterload such as hypertension or aortic stenosis. (iii) Microvascular dysfunction, which may actually be common even in patients with NOCAD [78,136,183,184,185,186,187].

#### 3.1.3. Imaging Tips

Both CMR and CCTA exhibit many advantages for algorithms of ischemia management [188].

CCTA plays a key role in the discrimination of obstructive and non-obstructive CAD patients with chest pain, or in detecting unprotected CAD (i.e., obstructive left main CAD) [189].

Given its negative predictive values close to 100%, CCTA plays a primary role in ruling out CAD disease for chest pain (for which it is not possible to exclude a cardiac genesis) in low-to-intermediate Bayesian probability CAD patients [78].

In this regard, CCTA has shown a relevant impact over recent years [190,191,192,193].

From the PROMISE study (a comparison between management of patients with suspected CAD by CCTA vs. traditional stress tests—i.e., excluding stress CMR), CCTA identified CAD better than stress testing, with a better prediction of cardiac events, especially in non-obstructive CAD. In the analysis by Hoffmann et al., a CCTA-derived low-risk group corresponds to an event rate of 0.9% over a two-year period vs. 2.1% observed in patients managed with the traditional stress test [194,195].

Similarly, from the SCOT-HEART trial (a comparison between management of patients with standard-of-care (SOC) vs. SOC plus CCTA), in patients with chest pain, there was a lower risk of death from CAD or non-fatal MI in patients managed with CCTA compared to SOC alone (2.3% vs. 3.9%/HR: 0.59, 95% CI: 0.41–0.84) [196].

Currently, CMR-derived cardiac imaging is effective for both definition of IHD and for ischemia detection, with important diagnostic and prognostic implications [197].

The “function-perfusion-tissue characterization” triad should be studied in IHD for an adequate evaluation of cardiac viability and ischemic burden.

**Figure 4:**
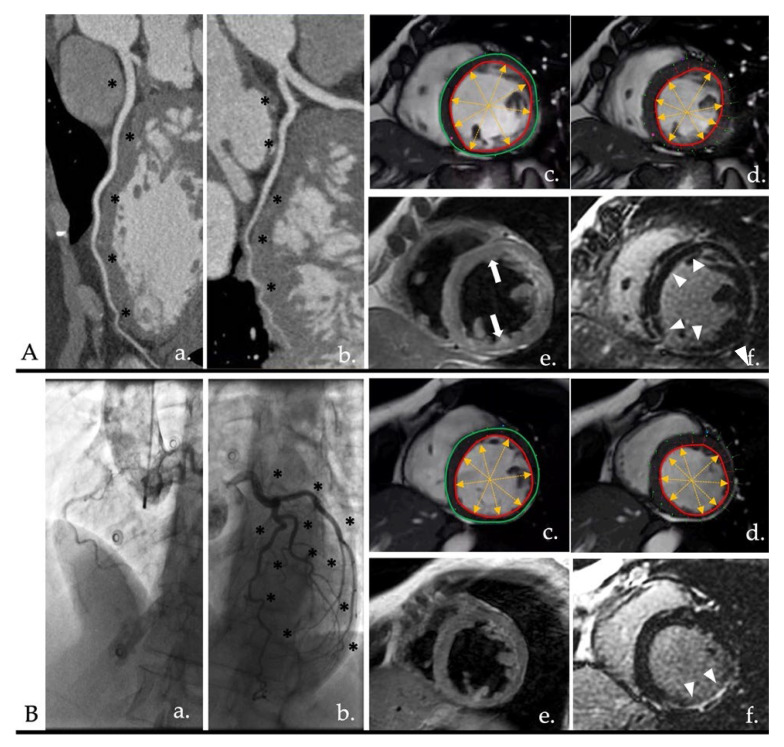
Panels (**A**,**B**) show the comparison of two patients with left ventricular dilatation and HFrEF. In images (**a**,**b**), anatomic evaluation of the coronary tree shows NOCAD (black asterisks). In images (**c**,**d**), cine images of the diastolic and systolic phase are shown, respectively. In both cases, the inferolateral wall appears to be thin. In panel (**B**) is evident a segmental akinesia compared to panel (**A**) which shows different areas of akinesia with a non-territorial distribution. Moreover, areas of fatty replacement could be highlighted in case (**A**) (image (**e**), white arrows), compared to case (**B**). Image (**f**) shows the LGE. Panel (**A**) shows a subepicardial and mesocardial LGE (non-ischemic pattern) with a ring-like distribution (>3 contiguous segment; white arrowheads). Panel (**B**) shows a subendocardial-to-transmural LGE (ischemic pattern). Panel (**A**) shows a case of DSP cardiomyopathy, while panel (**B**) shows a MINOCA-like syndrome. HFrEF: heart failure with reduced ejection fraction; NOCAD: non-obstructive coronary artery disease; LGE: late gadolinium enhancement; DSP: desmoplakin; MINOCA: myocardial infarction with non-obstructive coronary arteries.

As mentioned, the subendocardial distribution of LGE identifies an ischemic injury as opposed to fibrosis with meso- or subepicardial distribution, typical of non-ischemic alterations [110] (Figure 4).

CMR is also effective in defining myocardial viability through discrimination of LGE extension and segmental kinesis [198].

From the SPINS registry, extensive ischemic burden was related to a higher risk of major cardiac event, including hospitalization for congestive HF, and revascularization was associated with a protective effect only in the extensive ischemia subset [199,200,201,202,203,204,205].

Similarly, from the MR-INFORM RCT, stress CMR-related ischemia proved to be similar to FFR in stratifying ischemic patients, with no significant difference in MACE occurrence between stress CMR and FFR-harm [206].

Therefore, stress perfusion CMR or, similarly, the comparable CT-MPI should be performed for adequate risk stratification of IHD patients.

T2 sequences (both with T2 mapping and STIR) identify myocardial edema in acute injury, also allowing the definition of the area at risk as the difference between necrotic and more extensive edematous areas [207].

Lastly, both CMR and CCTA are capable in identifying ventricular thrombus in the adverse remodeling stage after infarction, as in aneurysmatic areas.

### 3.2. Dilated Cardiomyopathy (DCM)

DCM is a major cause of HF, and shows heterogeneous distribution among age, sex, and ethnicity [159].

From the study of Shore et al., in a cohort of 156,013 HF patients, DCM was present in 31% of cases. DCM is associated with a high prevalence of advanced HF and is among the leading causes of transplantation and cardiac death [208].

DCM can recognize different substrates, among which the genetic type recognizes some phenotypes with more aggressive evolution, including titin and laminin mutation. Recently, DSP mutation has been introduced among the dilated phenotypes, which is also common to some aggressive arrhythmogenic phenotypes [209,210].

Other common causes can derive from myocarditis, systemic inflammatory diseases or cardiotoxicity during chemotherapy [159,211].

The natural evolution tends to be variable with a mortality rate close to 77% over 2 years. Structural and functional recovery after an incidental finding of HF is possible only when the acute injury is not enough to cause extensive loss of myocardium. A recovery of remodeling reduces mortality by 50% at 10 years [212,213,214,215,216].

#### Imaging Tips

DCM is determined when CAD or conditions of increased pre-load are excluded.

CCTA could play an important role in excluding obstructive CAD from other DCM causes.

CMR, conversely, is the best imaging tool for DCM characterization, both for volume definition and tissue characterization. In this regard, LGE is an important unfavorable prognostic factor.

LGE may reveal non-ischemic patterns of enhancement, although the latter may be present under several conditions, thus being non-specific. Duchenne dystrophy, myocarditis and sarcoidosis can all present with LGE localized in the inferolateral wall of medium and basal planes, as often observed in arrhythmogenic CM when the left ventricle is involved [217].

Some imaging patterns, otherwise, could be more specific.

Laminin-correlated DCM could show a mid-wall septal LGE with a reported association with a non-compaction phenotype [218].

DSP, conversely, and aggressive filamin mutation, could show a typical non-ischemic ring-like LGE pattern, although this could result in higher arrhythmic burden compared to other DCM genotypes (Figure 4) [209,210].

Another important aspect is mitral regurgitation, which doubles the risk of mortality or worsening of HF in the presence of DCM [159].

**Figure 5:**
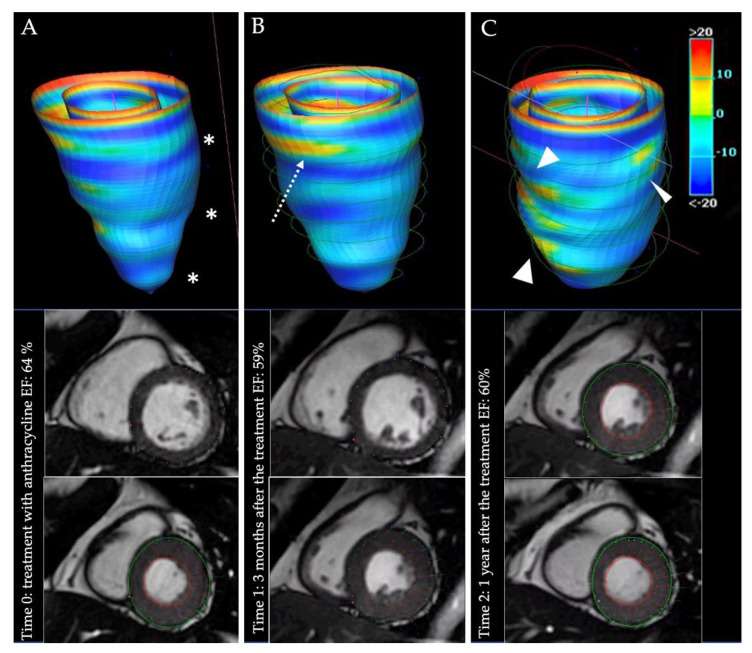
Panels (**A**–**C**) show the evolution of a cardiotoxic CHT with anthracycline. In panel (**A**), CMR examination obtained before the CHT. In panel (**B**,**C**), SA cine images of CMR examinations acquired at 3 and 12 months after the treatment. On the right, a volume rendered reconstruction obtained after contouring of entire SA stack, with a colorimetric map of peak strain. Colors correspond to the degree of deformation: brighter colors represent lower level of deformation. Similar kinesis and overall ventricular function are clearly preserved during the three examinations. In panel (**A**), overall longitudinal deformation is preserved (blue colors; white asterisks). In panel (**B**), the dotted white arrow highlights a lower deformation of basal segments compared to the first examination. Finally, after 12 months (panel (**C**)), peak strain analysis highlighted different areas of lower deformation (white arrowheads) and overall brighter colors meaning a reduced strain. CHT: chemotherapy; CMR: cardiac magnetic resonance; SA: short axis.

Lastly, mapping and strain imaging are also advantageous in detecting early myocardial injury during cardiotoxic chemotherapy [219,220,221] (Figure 5).

### 3.3. Hypertrophic Cardiomyopathy (HCM)

Hypertrophic phenotypes vary, ranging from primitive phenotype to hypertrophic phenocopies (i.e., hypertrophic mimics) [222]. Depending on the clinical conditions determining hypertrophy, the association with HF also changes.

Primitive HCM is associated with the mutation of myofibrils and sarcomere architecture. These alterations require increased energy to ensure adequate isokinetic tension, inducing disarray and myocytic hypertrophy. With the increased oxygen demand, these alterations result in ischemia, further determined by the microvascular alterations induced by myocardial hypertrophy. Moreover, these alterations may also have cumulative effects in determining myocardial fibrosis, adverse remodeling, and clinical evolution toward HF [159]. Nevertheless, thanks to a better knowledge of the pathophysiological aspects and natural evolution of HCM and an improved therapeutic approach, the risk of lethal complications has been extremely downsized, reporting a life expectancy superimposable to a non-affected population (mortality rate 0.5% per year, independent of age) [223].

On the other hand, storage diseases account for many hypertrophic phenocopies. On the spectrum of hypertrophic phenocopies, cell and matrix volumes increase proportionally in terms of health and disease; with increasing hypertrophy, both matrix and cell volume increase, apart from storage diseases, which are dominated by matrix expansion deriving from the storage of specific substances [224].

Primitive HCM is often associated with diastolic dysfunction and HFpEF, only leading to HFrEF when advanced stages of adverse remodeling occur (burned-out phase).

Less frequently, acute HF can be observed in phenotypes associated with arrhythmias, which can precipitate the symptoms.

Other factors can lead to HF: (i) Left ventricle outflow tract obstruction, with an annual risk for progression of 3.2–7.4%, while mid-ventricular obstruction is associated with advanced HF and an increased risk of mortality [225,226,227,228]. (ii) Adverse remodeling up to the “burned-out” stages, which are associated with 11% mortality, although they represent only 3% of presentations [229,230,231,232]. (iii) Increased atrial volumes [225,226,233].

Hypertrophic phenocopies often present as diastolic dysfunction. Anderson–Fabry disease (AFD) typically presents as HFpEF, further exacerbated by the presence of LGE and mitral regurgitation [234,235,236].

Finally, Danon disease typically manifests as HFrEF [237].

#### Imaging Tips

CMR is an uncontested imaging tool for HCM and other hypertrophic phenocopies. CMR indeed allows the morphological and functional characteristics of HCM to be defined, including LV wall thickness and hypertrophic distribution, ventricular function, valvular impairment, and myocardial fibrosis, overcoming the technical limitations of echocardiography [102,238].

**Figure 6:**
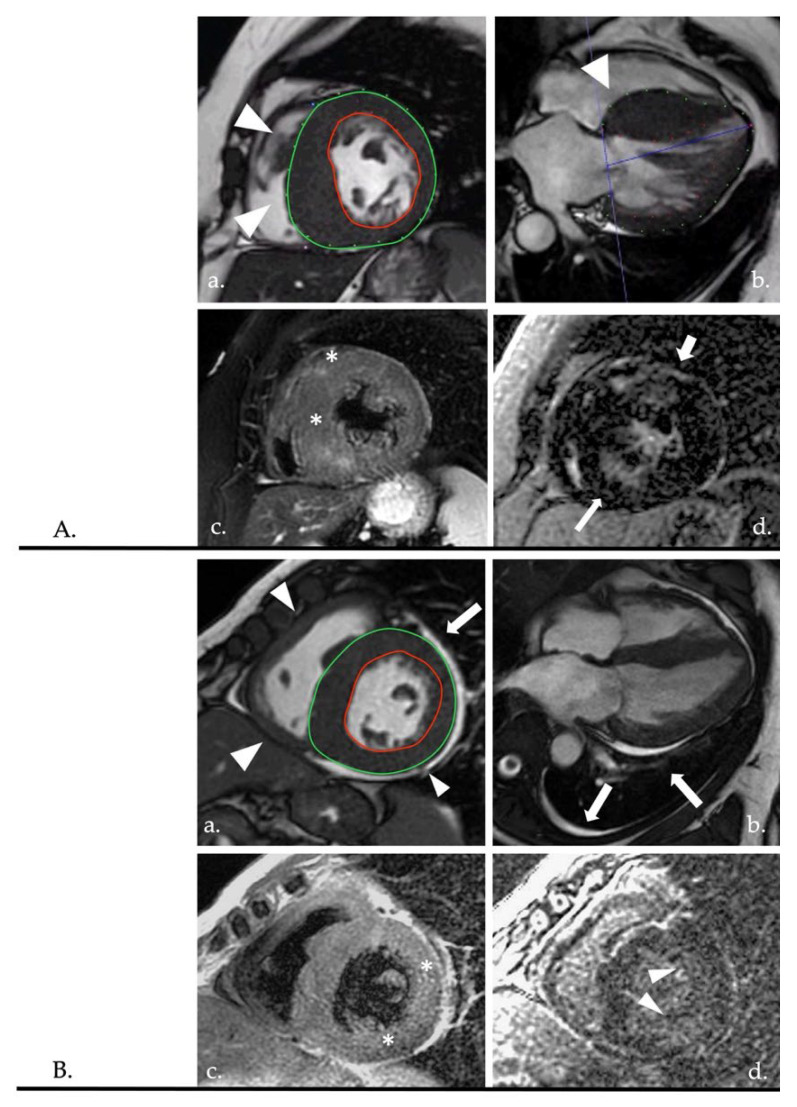
Panels (**A**,**B**) show two cases of HFrEF and myocardial hypertrophy. In panel (**A**), asymmetric hypertrophy is highlighted with prevalent involvement of the septum (images (**a**,**b**) showing frame of cine SA and HLA views). Patchy areas of edema (white asterisks) and LGE (white arrows) (images (**c**,**d**)) are clearly evident in the context of hypertrophic segments, with LGE also extended to non-hypertrophic segments (thick white arrow). Panel (**A**) findings are indicative of a primitive HCM. In panel (**B**), biventricular hypertrophy (white arrowheads in image (**a**) showing a cine-frame of SA view). Image (**b**) showing a cine-frame of the HLA view with evidence of pericardial and pleural effusion (white arrows). Image (**c**) shows a T2-w sequence with evidence of faded myocardial edema (white asterisks). Image (**d**) shows an LGE sequence, with the absence of clear myocardial nulling and prevalent subendocardial enhancement (white arrowheads) with an apparent transmural distribution in the inferolateral basal segment. Compared to panel (**A**), panel (**B**) findings clearly diverged from a typical primitive HCM pattern. Panel (**B**) findings indeed refer to a hypertrophic mimic (non-treated AL type amyloidosis). HFrEF: heart failure with reduced ejection fraction; SA: short axis; HLA: horizontal long axis; LGE: late gadolinium enhancement; HCM: hypertrophic cardiomyopathy; AL: amyloid light chain.

Different patterns of LGE and CMR mapping are important markers for differential diagnosis. Typically, a patchy distribution of LGE could be observed in primitive HCM. Conversely, absence of myocardial nulling could be more specific to cardiac amyloidosis, which can also mimic HCM, although myocardial amyloid accumulation results in restrictive physiology [239,240] (Figure 6). Moreover, abnormal T1 mapping values turned out to be specific for AFD (very low values indicative of intracellular adipose deposition) [241,242].

ECV fraction also seems promising in dichotomizing the myocardium into cell and matrix components for hypertrophic characterization [224].

In fact, although CMR, beyond other techniques, could provide some specific markers useful for a proper differentiation between different CMs (AFD can be suggested by unique features on CMR), multimodality imaging including nuclear imaging is necessary for the accurate non-invasive diagnosis of infiltrative HCM mimic. In this regard, Tc-99 m PYP proved to be effective in the identification of patients with the ATTR subtype (see Section 3.4) [243].

Edema with abnormal T2 findings could be observed in primitive HCM often indicative of an acute myocardial injury (i.e., ischemic extravascular damage) and associated with electrical instability [244].

Lastly, GLS provides more adequate prognostic information than EF as a more reliable global predictor beyond focal damage, revealing that HCM and other phenocopies are more prone to developing HFrEF, with a systolic dysfunction and preserved EF, which likely participates in the progression of disease [102].

### 3.4. Restrictive Cardiomyopathy (RCM)

RCM is one of the less frequent heart diseases [245].

Different phenotypes can be classified into (i) non-infiltrative RCM (idiopathic RCM, scleroderma, pseudoxanthoma elasticum); (ii) infiltrative (amyloidosis, sarcoidosis, Gaucher’s, Hurler’s); (iii) storage diseases (AFD, glycogen storage, hemochromatosis, and iron overload); (iv) endomyocardial (endomyocardial fibrosis (EMF), radiation-induced, drugs, carcinoid, metastatic tumor) [246].

RCM is usually characterized by regular-to-upper limit parietal thicknesses. However, in storage diseases, there may be a variable degree of myocardial thickening, overlapping with hypertrophic phenocopies and with evolution to dilation [245,247,248].

Despite the low frequency, RCM shows a high prevalence of HF, estimated at 83%, with poor prognosis and high mortality [249,250].

Primitive RCM derives from high cellular stiffness, related to the increased sensitivity of myofibrils to calcium with collagen deposits and intracellular aggregates.

RCM and reduced stroke volume can be associated with orthostatic hypotension and possible hypoperfusion if associated with hypovolemic episodes [159,251,252].

In the secondary RCM phenotype, on the other hand, the increased stiffness derives from the accumulation of the specific substance and the fibrosis that can result [159,251,252,253].

Among secondary phenotypes, hemochromatosis typically manifests as RCM, with iron storage causing changes in the transmembrane flow of calcium and diastolic dysfunction (HFpEF), loss of myocytes and fibrosis (cytotoxic effect).

Although hemochromatosis begins as HFpEF, natural evolution turns toward HFrEF and ventricular dilation.

Amyloidosis, instead, is more prone to developing into HFrEF, although in different ways depending on the etiology. Transthyretin (ATTR) amyloidosis tends to show a more aggressive phenotype, although amyloid light chain (AL) phenotype shows a more aggressive clinical evolution, exhibiting a 5-year survival of less than 10% [254,255].

The prognosis of HF in RCM is poor, regardless of the underlying cause of RCM [256]; e.g., five-year survival of thalassemia with HF is less than 50%. Moreover, HF has been identified as an important predictor of mortality in patients with sarcoidosis, with an expected 10-year transplantation-free survival of only 53% in individuals with overt HF [159,257,258,259,260].

#### Imaging Tips

Typically, CMR reveals reduced stroke volumes in primitive RCM, deriving from impaired ventricular filling and inadequate pre-load. Other typical findings are high atrial volumes with/without non-ischemic LGE [246,261].

CMR shows different advantages in cardiac amyloidosis. Specifically, analysis of segmental kinesis and strain can highlight prevalent involvement of septum and relative apical sparing. T1 and ECV can show a marked increase in T1 values and expansion of extracellular volume. LGE, moreover, can show the absence of myocardial nulling and diffuse subendocardial enhancement [262]. Finally, T2 mapping shows a significant prognostic implication in amyloidosis, resulting as increased, especially in non-treated AL phenotypes [263].

Multimodality imaging is particularly relevant in amyloidosis and sarcoidosis. A positive bone scintigraphy due to marked cardiac accumulation in the absence of serum or urinary evidence of free light chain (FLC) diagnoses cardiac amylogenic ATTR, even in the absence of histological evidence. In case of FLC findings, cardiac imaging using CMR or US can be used as a useful tool to demonstrate cardiac amyloidosis; however, cardiac or extracardiac biopsy is recommended for confirmation of AL type. However, some forms of amyloidosis AL and ATTR can coexist, so adequate typing by histology is recommended, generally using endomyocardial biopsy [239,240,264,265,266,267].

Similarly, CMR may also be quite effective for detecting sarcoidosis through granulomatous identification with LGE, more specific in subepicardial anteroseptal and inferolateral basal wall; however, diagnosis is difficult as its diverse features are often superimposable to other acute inflammatory and non-inflammatory injuries, and novel hybrid PET-CMR may help identify active granulomatous lesions with high sensitivity [268,269].

T2* mapping plays a pivotal role in defining hemochromatosis. The cut-off values for diagnosis of cardiac hemochromatosis are 20 ms, while 10 ms turns out to be significant as the worst prognostic factor, with values <10 ms being associated with an increased risk of development of HF or arrhythmias [270]. 

## 4. Conclusions

HF has become a major health problem, although it remains difficult to find a proper definition of this manifestation.

Advanced cardiac imaging plays a key role in defining primary cardiac injury in HF patients.

Notably, CMR facilitates in vivo virtual modeling, capable of providing important diagnostic and prognostic information.

CCTA, on the other hand, plays a marginal role, limited to the ruling out of CAD in patients with a low-to-intermediate risk. However, all potential applications deriving from current research will probably maximize its role in the coming years.
